# Supplementary material for: Effects of Group IVA Elements on the Electrical Response of a Ge2Se3-Based Optically Gated Transistor
Source: Micromachines (Basel). 2024 Aug 1;15(8):1000. doi: 10.3390/mi15081000 (PMC11356495; doi:10.3390/mi15081000)
Supplement: Supplementary file 1 [file micromachines-15-01000-s001.zip › micromachines-3116152-supplementary.pdf]

Supplementary Material for:

Effects of Group IVA Elements on the Electrical Response of Ge_2Se_3 Based Optically Gated Transistor

Md Faisal Kabir and Kristy A. Campbell *

Electrical and Computer Engineering, Boise State University, Boise, ID, USA
* Correspondence: kriscampbell@boisestate.edu

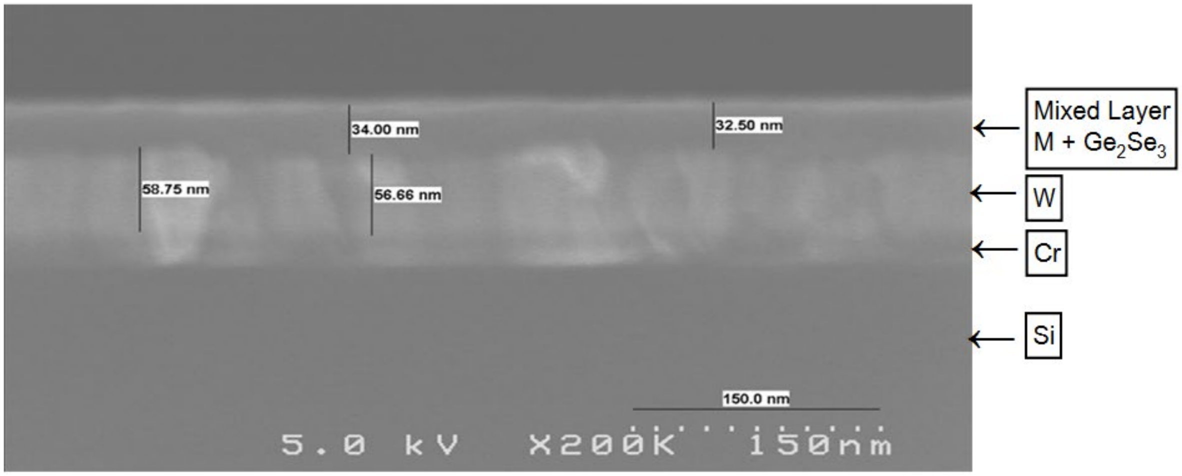

Figure S1. SEM image of the in-situ sputtered gate film. The deposition substrate is p-Si/Cr/W.

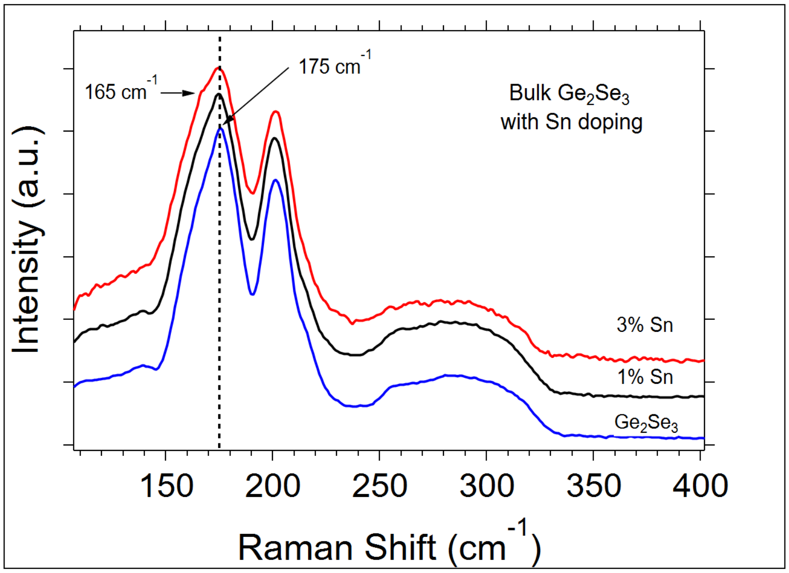

Figure S2. Raman spectra for Sn-doped bulk Ge_2Se_3.

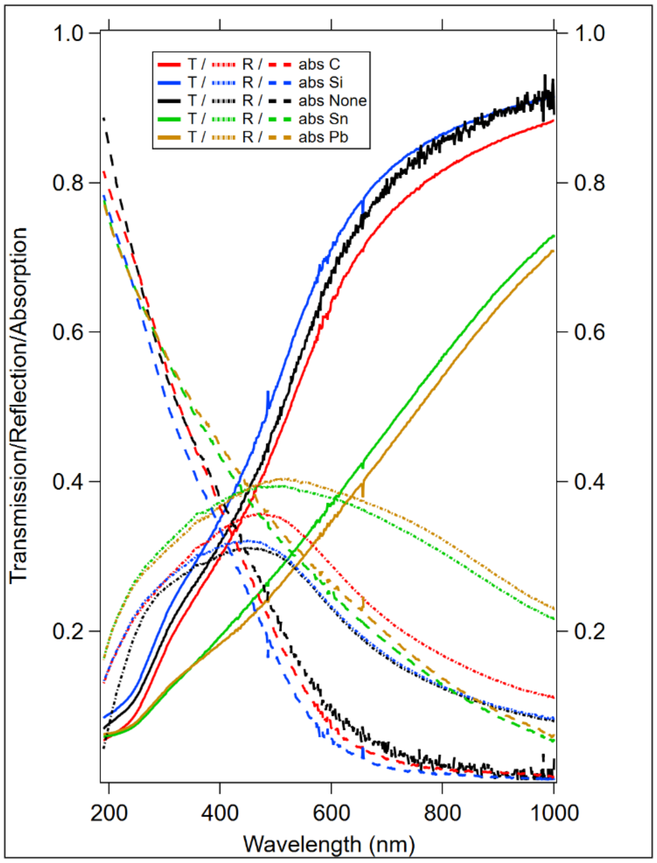

Figure S3. Reflection (R) measurements for each sample, along with transmission (T), and absorption (abs) calculated using R + T + abs = 1.

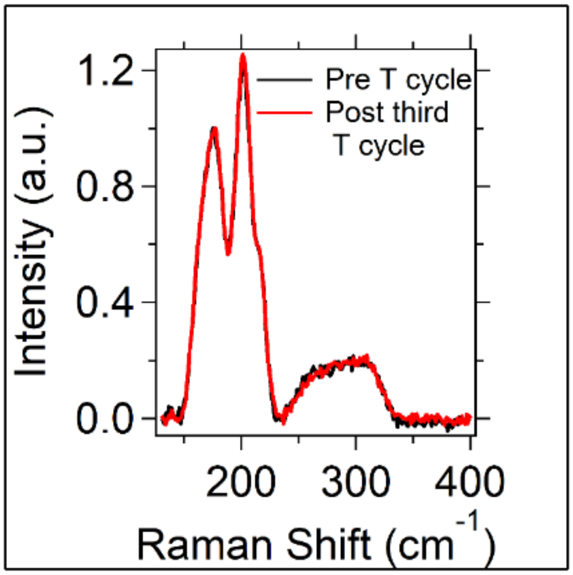

Figure S4. Raman spectra of the undoped Ge_2Se_3 measured before and after three temperature cycles from 20 to 140 °C.

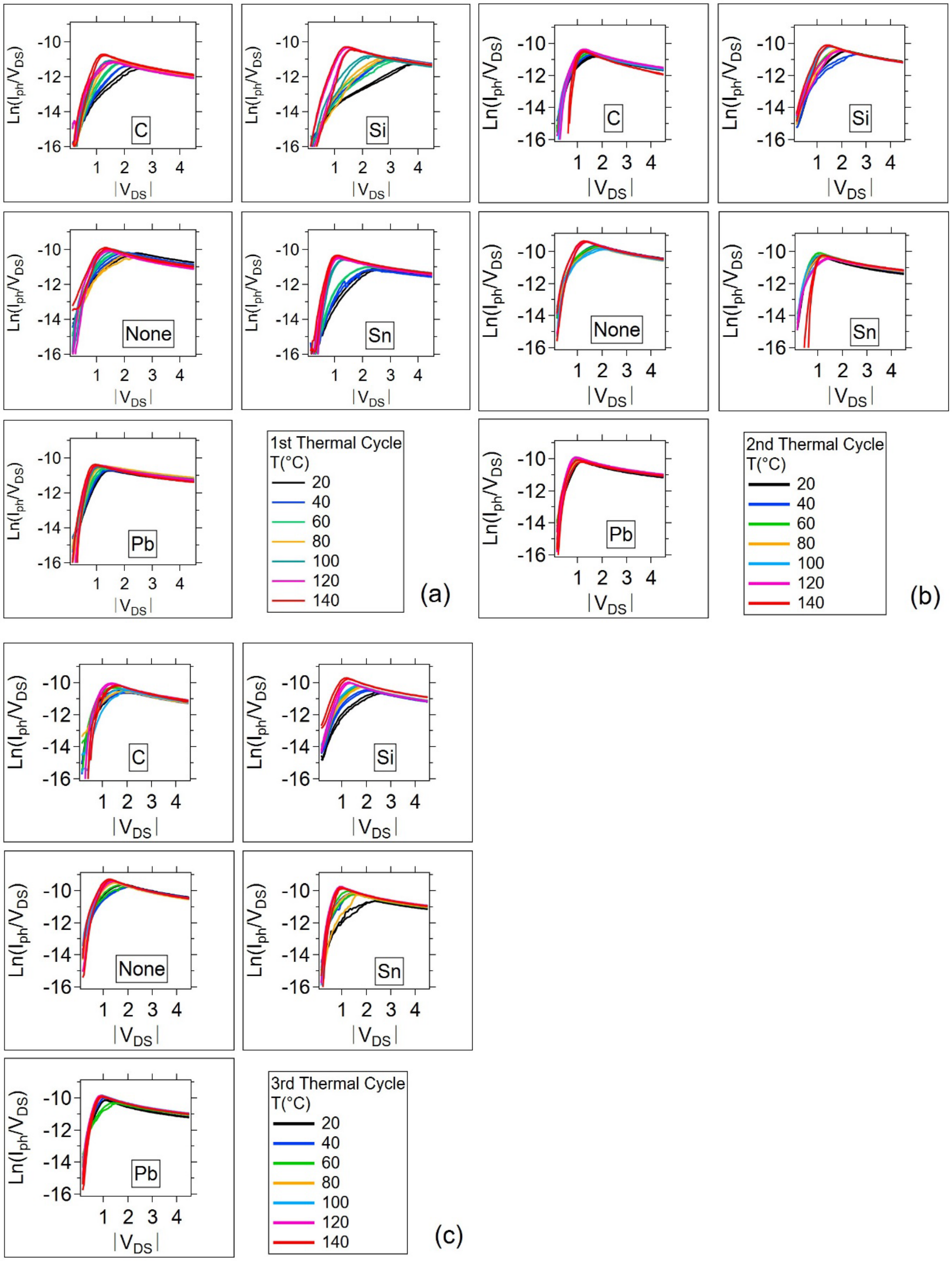

Figure S5. Ln(I_{ph}/V_{DS}) vs V_{DS} plots for all temperatures, all samples. (a) 1st thermal cycle; (b) 2nd thermal cycle; (c) 3rd thermal cycle.
